# Lateral Release – A technique for medium to large scalp lesions

**DOI:** 10.1111/ddg.70008x

**Published:** 2026-02-28

**Authors:** Julian Kött, Daniel Czesla, Moritz Felcht

**Affiliations:** ^1^ Department of Dermatology and Venereology University Medical Center Hamburg‐Eppendorf Hamburg Germany; ^2^ Fleur Hiege Center for Skin Cancer Research University Medical Center Hamburg‐Eppendorf Hamburg Germany; ^3^ Center for Dermatologic Surgery St. Josefskrankenhaus Heidelberg Academic Teaching Hospital of the Medical Faculty Mannheim Heidelberg University Heidelberg Germany

## INTRODUCTION

Skin tumors of the scalp are increasing significantly as the population ages, with basal cell carcinoma and cutaneous squamous cell carcinoma being particularly prevalent.^1^, ^2^ Large lesions that require extensive tumor excision often lead to defects that exceed the capabilities of simple reconstruction techniques. In addition to the size of the lesions, the reduced elasticity of the scalp and its convexity pose a particular challenge. Established procedures include techniques such as stretching plasty, rotation flap plasty, rotation flap plasty from two sides, transposition flap plasty, A‐T flap plasty, H flap plasty, secondary wound healing with subsequent split‐thickness skin grafting, partial closure and supplementary full‐thickness skin grafting, or free vascularized flap plasty.^3^, ^4^, ^5^

One way to reduce the tension of the suture is to make incisions in the galea next to the defect, which allows larger defects to be closed primarily.^6^, ^7^ Russo describes his experience with 119 defect closures on the scalp with defect sizes < 1 cm, 1–2 cm, 2–3 cm, 3–4 cm, and > 4 cm.^7^ A laterally removed incision was used in 22/33 of the 1–2 cm defects. Two lateral incisions were used in 14/21 defects measuring 2–3 cm and three lateral incisions in 7/14 of the 3–4 cm lesions.^7^ We would like to present this technique to JDDG readers and explain our surgical approach.

## TECHNIQUE

Lateral Release is based on a central stretch plasty, supplemented by one or two lateral incisions. The incision must be sufficiently deep and include the galea aponeurotica, as this is what most hinders mobilization. In our opinion, this technique is a good procedure for scalp defects measuring 3–4 cm.

**FIGURE 1:**
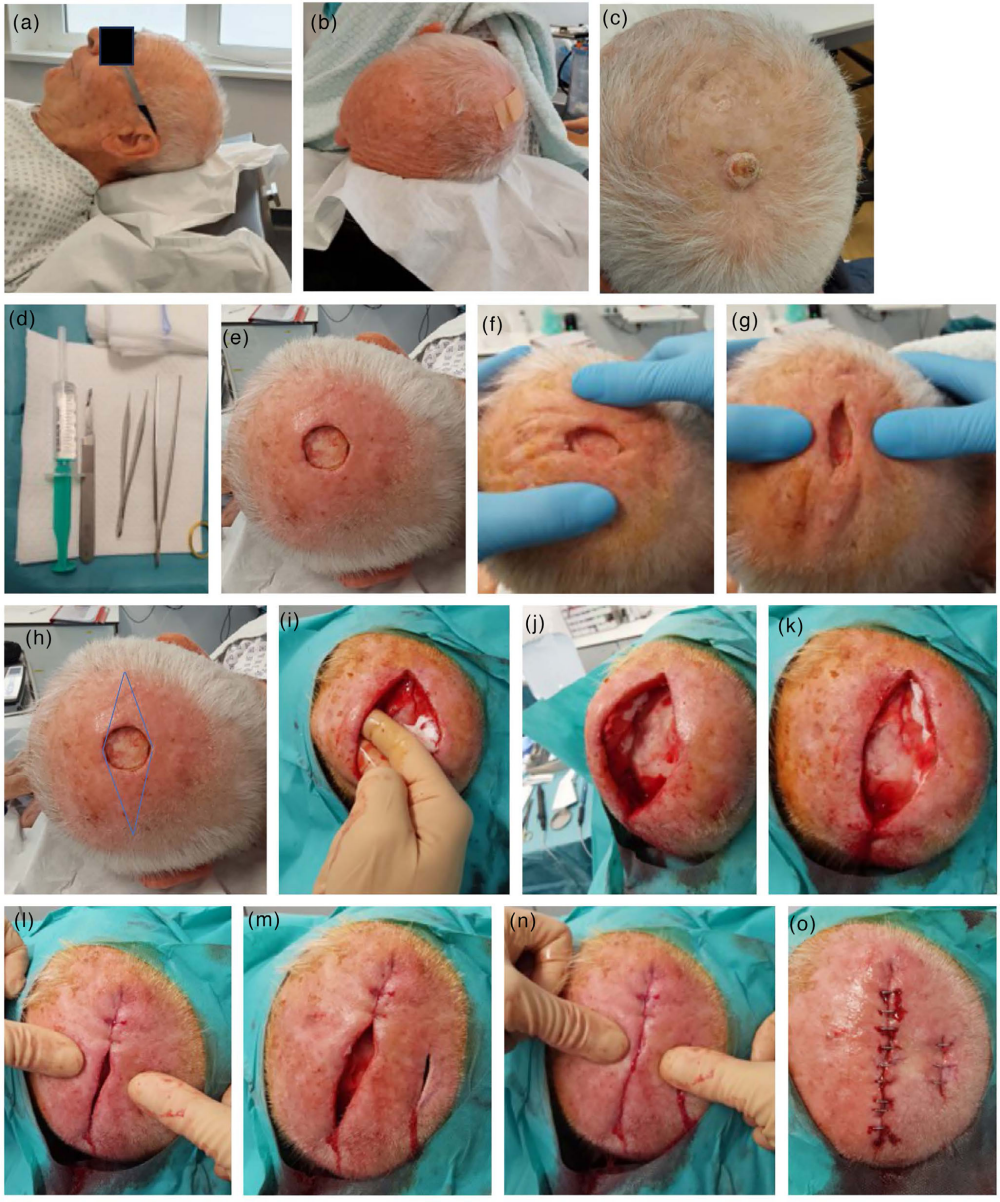
Detailed illustration of the lateral release technique. (a–b) The patient is positioned horizontally. (c) Excision of a squamous cell carcinoma. (d) Before the first incision, an additional injection of sterile local anesthetic is administered. (e–g) Creation of a defect measuring 4 × 4 cm. (h) The pinch test demonstrates the greatest skin mobility in the cranial and caudal directions. (i) Marking of the compensation triangles to utilize the areas of greatest mobility. (j) Blunt dissection using the fingers. (k–l) Insertion of sterile compresses into the prepared wound pockets. (m) Primary wound closure as far as possible without tension. (n) A lateral release incision is made caudal to the defect. (o) Tension‐free closure is achieved. (p) Complete wound closure using staple sutures.

1. *Positioning*: For all deep procedures on the scalp, we recommend a horizontal position (or, if necessary, a lateral position for findings at the back of the head) to avoid air embolism in a vertical position, as described in the literature (Figure 1a, b).^8^ The pathophysiology of air embolism is not yet fully understood and the incidence is very low. Other surgeons prefer different positions to avoid the increased tendency to bleed in the horizontal position.
2. *Complete excision of the tumor* (Figure 1b–d). It should be noted that surgical procedures on the skull are perceived as particularly painful despite significant amounts of local anesthesia. It can be assumed that this is due to particularly good absorption of the local anesthetic. A typical vicious circle that must be avoided is that pain leads to an increase in blood pressure, which in turn leads to increased bleeding. This requires more intensive electrocautery hemostasis, which further intensifies the pain symptoms. For this reason, it is helpful to inject sterile local anesthetic again before the first pain stimulus (usually the incision) (Figure 1d). We routinely use ibuprofen for postoperative analgesia.
3. The *Pinkus pinch* test is used to check which sides of the skin can be mobilized most easily (Figure 1f,g).^9^
4. As is usual in stretch plasty, *30° compensation triangles* are selected in order to distribute the tension as widely as possible (Figure 1h, blue lines). This can be done with a monopolar knife in order to *(1)* work as quickly as possible and avoid the vicious circle of pain described above, and *(2)* directly obliterate all smaller vessels of the subdermal plexus.
5. *Extensive blunt mobilization in the subgaleal space* (Figure 1i). It is essential that the dissection is performed in the correct layer. Dissection on the superficial musculoaponeurotic system (SMAS) leads to severe bleeding, and the necessary hemostasis sometimes results in damage to the subdermal plexus, which can lead to necrosis. During dissection, you can tell whether you are in the correct layer if *(1)* there is little bleeding and *(2)* blunt detachment of the galea from the periosteum, using scissors or your hand, produces a characteristic crunching sound. After preparation, it may be helpful to tamponade the newly created wound cavity with a compress (Figure 1j,k). This is intended to pre‐stretch the skin before closure and make the wound edge more accessible for necessary hemostasis.
6. After further mobilization on both sides and effective hemostasis, *closure* is performed with deep high corium sutures, as far as this is possible without tension. A residual area remains in the center that cannot be closed (Figure 1l).
7. One or, if necessary, two deep relaxation incisions (*lateral release*) are made on either side of the defect, *parallel to the main axis* of the stretching procedure: These are made centrally and are usually no longer than the original defect length (3–4 cm). Shorter incisions (e.g., 80 % of the defect length) are preferable (Figure 1l). It is important that the distance between the defect and the lateral release incision is sufficiently large and should not be shorter than the length of the newly created additional incision. For example, if the incision is 3 cm long, the distance between the lateral release incision and the defect should also be at least 3 cm. In particular, the incision should be deep enough to *cut into the galea*. This is the only way to achieve additional mobility. If necessary, further subgaleal mobilization via the lateral release access points may be required.
8. Now the central residual portion of the *primary defect can be closed without tension* (Figure 1n,o).
9. The Lateral Release incision(s) is/are then closed with corium sutures, skin sutures, or staples (Figure 1o).

Lateral release and the corresponding lateral incision(s) (one or two) enlarge the area to be undermined, thereby increasing mobility and enabling closure of the defect. This is achieved by incising the least mobile layer, the galea aponeurotica.

Limitations of the technique include sizes greater than 5 cm, fibrosis or scarring due to radiation or previous surgery, and the associated reduction in elasticity and mobility. The following risks must be taken into account: bleeding, secondary bleeding, and infection in general; in addition, occipital neuropathy depending on the location.

## DISCUSSION

**FIGURE 2:**
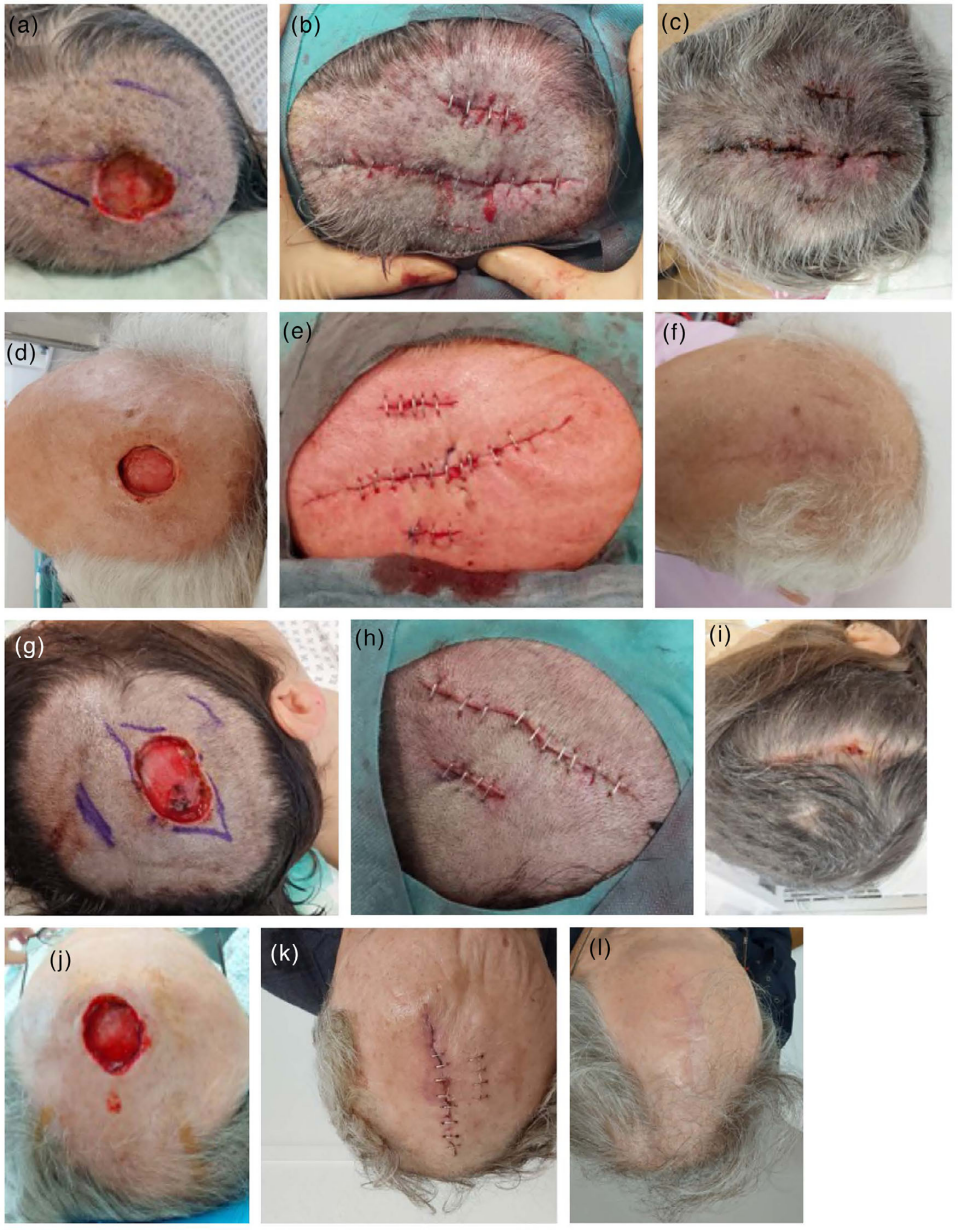
Clinical examples of the lateral release technique (a) Scalp defect (2.8 × 3.8 cm) after in sano excision of a lentigo maligna melanoma with lateral release performed on both sides (b) Postoperative result after closure with staple sutures (c) Two weeks postoperatively, removal of the staples reveals irritation‐free scarring (d) Scalp defect after excision of a lentigo maligna melanoma (tumor thickness 0.4 mm; defect size 4 × 4 cm) (e) Closure using two lateral release incisions (f) Good postoperative result at 3 months (g) Scalp defect after excision of a solid basal cell carcinoma arising in a nevus sebaceus (defect size 5 × 3 cm) (h) Closure using a stretch plasty combined with a lateral release incision (i) Good postoperative outcome at 8 weeks (j) Scalp defect after excision of an ulcerated basal cell carcinoma with perineural invasion (defect size 3 × 2.5 cm) (k) Closure including a lateral release incision (l) Satisfactory postoperative result at 6 months.

**FIGURE 3:**
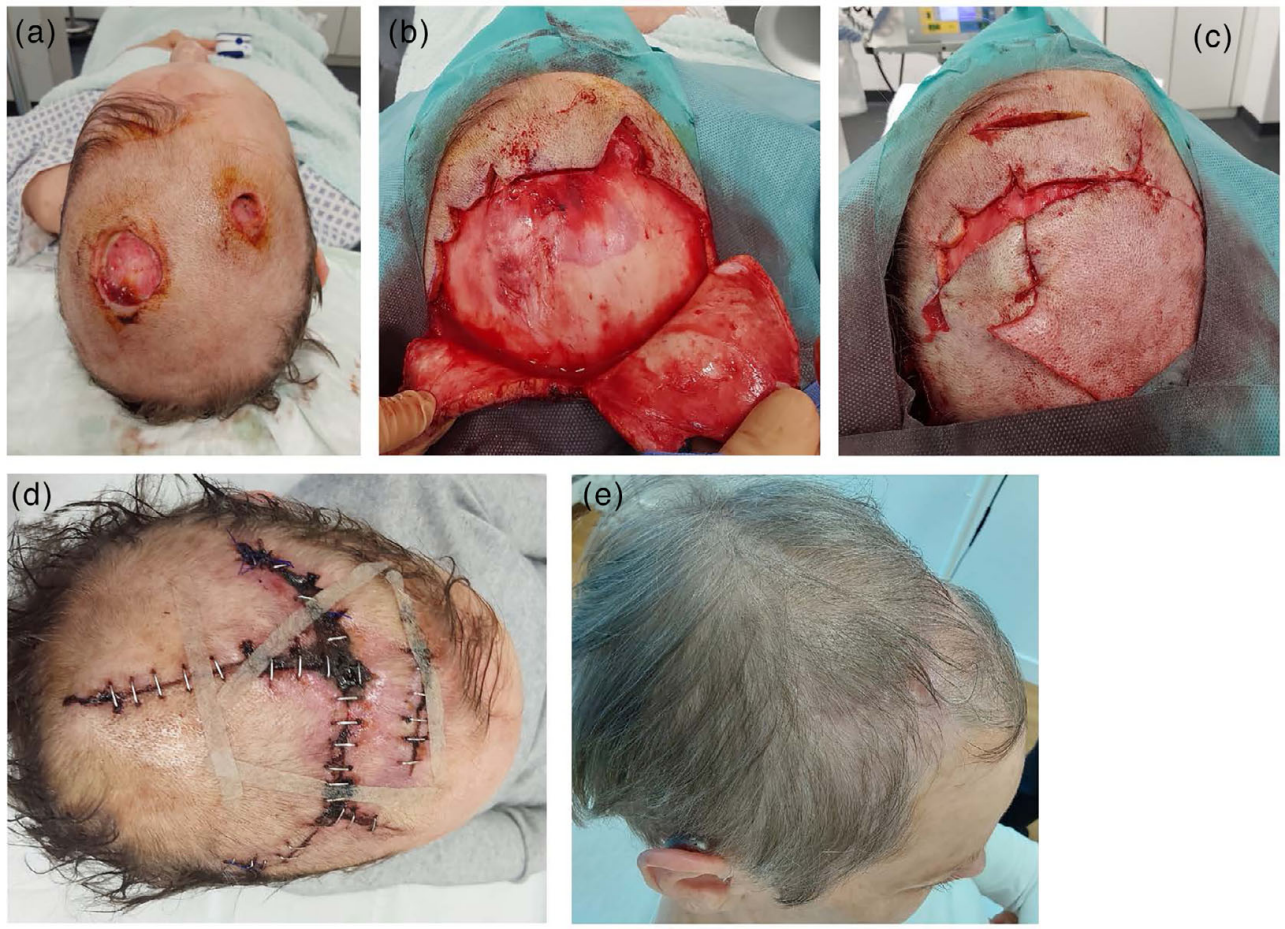
Clinical example of a lateral release incision in two rotation flap plasties (a) Scalp with two large defects following recurrence of a multilocular infiltrative solid basal cell carcinoma in the left parietal region and a multifocal solid basal cell carcinoma in the right parietal region. (b–e) Closure was achieved using two rotation flap plasties, six compensatory triangles, two back cuts, and one lateral release incision on the forehead, resulting in an acceptable postoperative outcome after 5 months.

Lateral release is a simple and effective method for closing large defects in the scalp after tumor excision. Targeted tension redistribution enables primary wound closure, which is aesthetically and functionally convincing (Figure 2). This technique is ideal because it can be performed with minimal material and time expenditure. The advantages include reduced tension in the central area of the wound, shorter operating time compared to complex reconstruction techniques, and good functional and aesthetic results. Disadvantages of capillitium procedures include pain despite high doses of local anesthetic (see above), possible injury to veins at the relaxation incisions, and the rare but serious complication of air embolism. The authors generally use lateral release in stretching plasty (Figure 2). However, it can also be helpful in combinations of rotational flap plasty (Figure 3).

Baker et al. describe the modified bipedicled flap for oncological defects on the trunk and extremities, which also features a lateral incision to reduce tension.^10^ Compared to the lateral release described here, the modified bipedicled flap is rounded and significantly longer than the incision on the actual defect. This reduces the tension on the defect suture but significantly increases the total defect area. The lateral incision with subsequent closure is also reminiscent of the keystone flap, which can usually be used on the lower extremities.^11^

Compared to rotational flap plasty, A‐T flap plasty, rotational flaps from two sides, transposition flap plasty, double rotational flap plasty, and H flap plasty, lateral release does not require extensive skin displacement or additional grafts.^3^, ^5^ Compared to secondary wound healing with subsequent split‐skin coverage, if necessary, it eliminates the need for subsequent surgery and minimizes the risk of infection through direct wound closure. In addition, the sometimes lengthy and costly wound care required for secondary wound healing is no longer necessary. Another surgical solution would be partial closure in combination with secondary wound healing or split‐thickness skin grafting of the central defect at a later stage.^12^ The disadvantages here include numerous dressing changes, the risk of infection with an open defect, and the visible scar from an aesthetic point of view. The time saved during surgery can be cited as an advantage. In addition, partial closure in combination with a direct full‐thickness skin graft is possible, but this also has aesthetic disadvantages in the form of a permanent scar or graft significantly below the skin level and an additional defect on another part of the body that would also need to be covered.

## CONCLUSIONS

Lateral release offers a minimally invasive solution for closing large defects in the scalp. The technique is easy to learn, resource‐efficient, and offers good functional, aesthetic, and surgical results.

## CONFLICT OF INTEREST STATEMENT

None.
